# Impact of rising temperatures on the biomass of humid old-growth forests of the world

**DOI:** 10.1186/s13021-021-00194-3

**Published:** 2021-10-12

**Authors:** Markku Larjavaara, Xiancheng Lu, Xia Chen, Mikko Vastaranta

**Affiliations:** 1 Institute of Ecology and Key Laboratory for Earth Surface Processes of the Ministry of Education, College of Urban and Environmental Sciences, Peking University, Beijing, China; 2 School of Forest Sciences, University of Eastern Finland, P.O. Box 111, 80101 Joensuu, Finland

**Keywords:** AGB, Biomass, Climate change, Forests, GPP, Respiration, Temperature, Warming

## Abstract

**Background:**

Understanding how warming influence above-ground biomass in the world’s forests is necessary for quantifying future global carbon budgets. A climate-driven decrease in future carbon stocks could dangerously strengthen climate change. Empirical methods for studying the temperature response of forests have important limitations, and modelling is needed to provide another perspective. Here we evaluate the impact of rising air temperature on the future above-ground biomass of old-growth forests using a model that explains well the observed current variation in the above-ground biomass over the humid lowland areas of the world based on monthly air temperature.

**Results:**

Applying this model to the monthly air temperature data for 1970–2000 and monthly air temperature projections for 2081–2100, we found that the above-ground biomass of old-growth forests is expected to decrease everywhere in the humid lowland areas except boreal regions. The temperature-driven decrease is estimated at 41% in the tropics and at 29% globally.

**Conclusions:**

Our findings suggest that rising temperatures impact the above-ground biomass of old-growth forests dramatically. However, this impact could be mitigated by fertilization effects of increasing carbon dioxide concentration in the atmosphere and nitrogen deposition.

**Supplementary Information:**

The online version contains supplementary material available at 10.1186/s13021-021-00194-3.

## Background

Forests influence climate change by storing C (carbon) that could alternatively be held in the atmosphere as CO_2_ (carbon dioxide) [1]. However, forests are also influenced by climate change, as temperatures impact tree physiology [2]. If this feedback is negative and more C is stored in forests with warming air temperatures, forests mitigate climate change. If the feedback after a time lag is positive, global warming will continue despite a complete cessation of direct anthropogenic emissions. Knowledge concerning the impact of warming temperatures on forests is necessary for quantifying global warming caused by a given anthropogenic emission level.

Multiple methodologies are available for empirically studying the impact of changing temperatures on forests. Perhaps the most straightforward conceptual approach is to simply document how growth has changed in recent decades and then assume the same trend to continue in the future. Canadian inventory data do not indicate a general change in trunk basal area growth [3]. Similarly, height growth in Finland seems to have increased very little or not at all [4]. In the Amazon rain forests, biomass growth increased in the 1990s but has since levelled off [5]. Biomass for a given tree density in fully stocked self-thinning plantations has increased in both Central and Northern Europe [6], indicating a modest but clear increase in productivity.

In addition to changing temperatures, other factors including changing CO_2_ concentration, N (nitrogen) deposition and changing precipitation have also influenced the long-term trends in tree growth. Disentangling their impacts is possible by focusing on year-to-year variability, e.g. on annually repeated diameter measurements based on tree rings or dendrometers. Diameter growth has been faster during warmer years in northern Finland [7] and northeastern United States [8]. On the contrary, diameters grew less in lowland Costa Rica during years with warmer diurnal minimum temperatures [9]. In line with these local studies, a global data set reported positive correlation between temperature and tree ring widths in the northern boreal but negative correlation in other biomes [10]. Even when focusing on year-to-year growth variation, the temperature signal may be masked by covarying meteorological variables, such as cloudiness, by other variables, such as investment in reproduction, or by time lags of years, e.g. if trees build reserves during good years instead of adding to their growth or if e.g. waterlogging kills deeper roots limiting growth over following years. These challenges can be avoided with long-term experiments. Warming all the tissues of large trees is practically impossible, but warming of the soil in a temperate forest increased biomass growth [11], and whole-plant warming of potted tropical trees had mixed impacts on their growth [12].

Results on tree growth are valuable but insufficient for understanding the forest-warming feedback for which stored C matters, as mortality is likely to change as well [13]. For example, if mortality increases more than growth, a warming-triggered boost in growth will lead to reduced biomass and C emissions into the atmosphere. Studies reporting changes in biomasses are therefore especially valuable. Unfortunately, most forests are typically successional, even in remote areas in biomes influenced by large-scale disturbances, such as fires, and are far from a hypothetical old-growth state in which biomass can be assumed to remain unaltered without global change impacts. However, a biomass change in remote regions or tropical rainforests lacking fires can be assumed to be naturally steady state, and any shift can be attributed to global change. Forests in the Amazon basin [5], equatorial Africa [14] and Borneo [15] have all been shown to increase in biomass, but as with studies regarding growth, disentangling the impact of warming temperatures from other global change drivers is difficult. Furthermore, measuring large trees is surprisingly challenging [16], and even minor inaccuracies may significantly bias subtle trends [17]. An additional challenge is that a seemingly stable (without global change) old-growth forest may be actually recovering e.g. from anthropogenic disturbances that occurred centuries ago or from more recent natural disturbances such as drought [18].

The above-presented empirical approaches have provided valuable views from numerous perspectives, but overall understanding unfortunately remains blurred, and even the direction of global forest biomass change caused by warming is questioned. Furthermore, all these empirical approaches neglect longer time lags in future biomass change, which are important in relation to precipitation [19]. For example, an increasing temperature may weaken and kill the dominant tree species adapted to colder temperatures [20] before a species adapted to the new, warmer climate is able to accumulate biomass above the initial level. If the new species were already present in the forest, the transitional phase could last for decades but would take longer if tree migration is involved. These challenges suggest that modelling may significantly improve our understanding concerning the long-term impacts of global warming on forest biomass from that based on empirical research.

Models on global biomass patterns range in how strongly they are based on physiological processes determining biomass rather than the direct statistical estimation of biomass. For example, at the statistical end of this continuum of modelling approaches, highest AGB (old-growth above-ground biomass) has been shown to be found in mid-latitude forests, but plenty of scatter remains unexplained both at global [21] and continental scale [22]. Such direct statistical modelling is not well suited for global future biomass modelling, as some future climates are currently found nowhere on Earth [23]. Models including physiological processes vary greatly in their complexity and in whether individual processes are determined theoretically or based on observations. Earth system models, which attempt to quantify all major processes in the ecosphere, are not good at predicting current AGB variation [17], which questions their ability to predict future AGB. Besides, their complexity complicates open discussion on their structure and assumptions. There is clearly a substantial need to model current global AGB and apply the same approach to predict long-term future changes with a simple physiological model. Our objective was to quantify changes in AGB caused by climate change in humid regions of the world based on a simple energetic approach that was able to explain well the current global AGB variation [24, 25] and to enumerate the causes of these changes.

## Methods

The normal way to understand biomass based on physiological processes is grounded on the production and decay of materials [26]. Based on this thinking, when focusing on a steady-state old-growth forest, exceptionally high biomass can result from large production or long residence time, i.e. turnover of the material produced, or both. The focal “material” ranges from focusing only on woody biomass [27] to all ecosystem C [28]. When applied to the AGB:

1 $$AGB=\frac{NPP}{D},$$

where NPP is above-ground net primary production and D is its decay, i.e. the reciprocal of turnover. NPP can be quantified with field measurements and understood based on physiological theories. However, even though D is intuitively reasonable, it is difficult to explain physiologically or make guesses about its climate-driven variation, unlike when focusing on dead tissue that decays predictably [29]. In general, D increases with decreasing size of trees [30], as they have a smaller proportion of biomass stored in long-term pools such as trunks [31]. However, plants also vary in how often they replace their leaves in a given climate. Deciduous trees have both higher NPP and D than evergreens, possibly resulting in the same AGB. Because of these challenges, in practice, D is obtained statistically as the quotient of NPP and AGB.

Perhaps less intuitive, but better for numerous reasons, is focusing on the input and output of energy, i.e. chemical energy in both structural and non-structural materials, instead of focusing on more permanent structures only. Maintaining living biomass consumes energy. As the input of energy in the ecosystem, GPP (gross primary productivity) does not vary much during succession after canopy closure [32], there must be a maximum biomass that the available energy can support and that can be estimated based on GPP and maintenance cost. In this energetic approach, Eq. 1 is modified to:

2 $${AGB}^{b}=\frac{GPP}{MCB},$$

where b is a parameter and MCB is the maintenance cost per unit biomass, not only including autotrophic respiration but also heterotrophic respiration resulting from the turnover of tree parts and mortality [24]. Parameter b was added, as autotrophic respiration does not increase proportionally with tree size [33], and was parametrised to 0.4 with a global data set [24, 25]. In practice, similarly as with D in Eq. 1, MCB is best understood as the quotient of GPP and AGB^b^, and this understanding can then be used to model AGB. MCB in Eq. 2 can be understood better based on temperatures than D in Eq. 1, as, in addition to heterotrophic respiration (that was challenging to model), it is composed of autotrophic respiration with a well-known temperature dependency [34]. Data on GPP are readily available from eddy flux towers and can be modelled straightforwardly based on temperatures.

Water is crucial for trees, and many global patterns are well explained by annual precipitation [19]. Its influence on AGB is certainly important [21] and probably increasing in importance [10]. However, it is challenging to incorporate water into AGB modelling, as trees may obtain water from deep layers of the soil [35], and therefore also a dry climate may therefore be conducive to tree growth. Even reaching the water tables of forested regions of the world seems to be biomechanically easy compared to building massive trunks upwards that have to resist winds and gravity. Instead of the direct impacts of water scarcity potentially leading to hydraulic failure or C starvation caused by inability to photosynthesise [36], much of the precipitation impact on AGB could be caused by the covarying vapour-pressure deficit or even more indirectly via wildfires or other disturbances that increase with dryness [37], further complicating the quantification of the mechanisms. These matters drove us to focus only on humid forests.

As the energetic temperature-based approach (Eq. 2) explained well the current global AGB variation in humid forests around the world [24, 25], it is ideally suited to approximate temperature-caused long-term future changes in the AGB of humid forests. To do this, we used historic monthly means of diurnal minimum and maximum temperature (near-surface air temperature) data from 1970 to 2000 of the WorldClim 2.1 data set [38]. We call this climate and the resulting AGB “current” for simplicity, even though some decades have passed. Because of time lags in AGB response to temperature, we intentionally use the term “current” to express this vagueness. To study the impact of warming temperatures on AGB, we used projected temperatures for 2081–2100, which were estimated based on an ensemble of models [39] and were run based on an intermediate emission scenario Shared Socio-economic Pathways (SSPs) and Representative Concentration Pathways (RCPs) (SSP2-4.5) [40]. Similarly, as with the “current” climate and AGB, we use the terms “future climate” and “future AGB” to refer to these predicted temperature regimes and predictions based on them. If the climate were to stabilise at this future climate, AGB would stabilise at its new level after a time lag once genotypes of the same species or new tree species adapted to the climate have become dominant. We used a 10-min spatial resolution in the analysis.

We decided not to modify the models or use updated parameterisation data sets, to allow interpretation of the results based on the earlier papers [24, 25] and to avoid overparameterisation. In this approach, GPP is primarily dependent on temperature that is assumed to cycle diurnally between the monthly mean minimum and maximum that occurs 4 h after solar noon, with coldest daytime temperatures at sunrise. GPP was parameterised to fit a global data set [25] and is positive between − 5 and + 40 °C, peaking at + 25 °C. This reflects the temperature dependence of the physiological processes involved [41]. The maximum of + 40 °C corresponds well to leaf temperatures at which tropical trees stop photosynthesising [42], even though temperatures leading to irreversible damage are some ten degrees higher [43]. However, well-watered trees can be significantly cooler than air temperatures [44], and our + 40 °C limit may be too low for tropical forests. Despite this, we assumed this dependency for all forests (but see the sensitivity analysis) but took adaptation and acclimation into account by cutting down GPP the greater the difference between mean temperatures of the month in question and the previous month. Therefore, GPP is higher during a month with a given temperature in a hypothetical climate without temperature variation than if the previous month was colder or warmer and trees therefore had not acclimatised perfectly to this given temperature. In addition, in this approach, GPP is only possible with positive sun angles (i.e. during daylight) and is higher with a higher angle.

AGB depends on the ratio of GPP and MCB (Eq. 1), and MCB is computed by assuming the so-called Q_10_ temperature coefficient, with a 1.67-fold MCB for an increase of 10 °C [25]. As with GPP, a change in temperature from the previous month and imperfect acclimatisation cause an extra energetic expense that is proportional to the temperature difference in mean monthly temperatures.

We excluded arid and high areas in the same way as the study fitting the physiological models [24]. We computed annual potential evapotranspiration based only on current temperatures with a classic method [45], and excluded areas where current annual precipitation was smaller than the potential evapotranspiration based on WorldClim 2.1 [38]. We excluded highlands at least 1000 m above sea level [46]. However, we did not exclude oceanic islands that have never been connected to continents, as was done previously [24], but as their total area is minimal, their impact on our results are also expected to be minimal. Theoretically, our findings on oceanic islands should be viewed as indicating the potential AGB of exotic continental tree flora in the climate of each island, rather than the AGB of the local depauperate species communities.

Thanks to the simplicity of the modelling approach, we could quantify the sensitivity of our results relative to all 13 parameters, parameter bounds or data selection criteria that influenced either the maximum temperature at which GPP is possible or the base of 1.67 on the temperature sensitivity of MCB. We were interested in this maximum temperature and the base number, as we expected these to drastically influence how increasing temperatures are expected to affect AGB, particularly in the tropics.

## Results

**Fig. 1:**
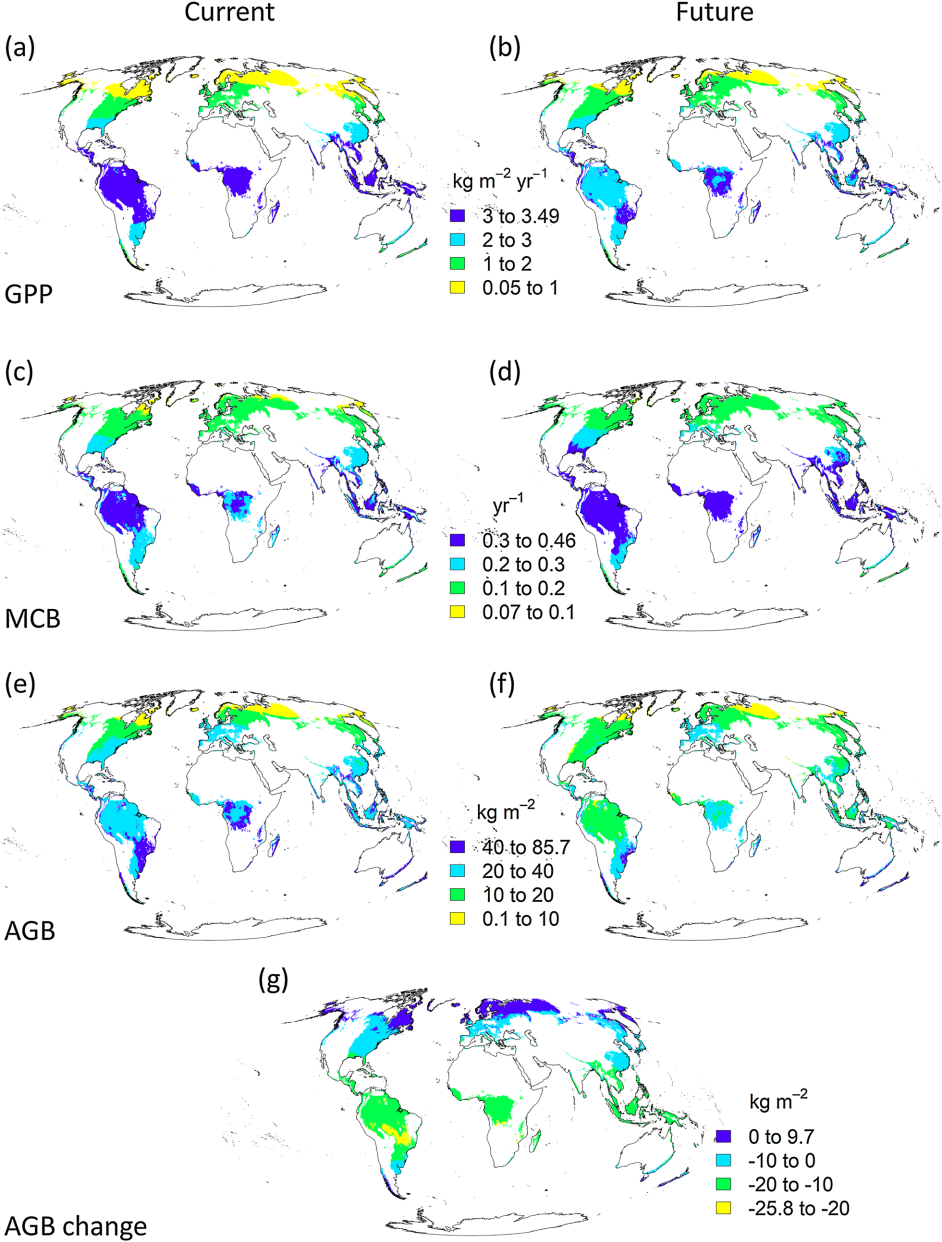
GPP, MCB and AGB currently based on the climate of 1970–2000, and future values based on a projected climate for 2081–2100

In the current climate (1970–2000), GPP (gross primary productivity) was estimated to be highest in the tropics (see Table 1 for definitions of latitudinal zones) and to decrease towards the poles (Fig. 1a). In the future climate (2081–2100), estimated GPP increased in all other latitudinal zones except the tropics (Table 1). However, due to the large area of humid lowland forests in the tropics, the global mean decreased slightly (Table 1). Increasing temperatures levelled the global variation in GPP, and the intermediate values expanded geographically (Fig. 1b). The 4.6-fold GPP in the tropics relative to the boreal dropped to a 3.2-fold difference.

**Table 1:** Four latitudinal zones, their areas, estimated GPP, MCB, AGB (old-growth above-ground biomass) and net C flux assuming that 47.6% of AGB is C [65] for the studied humid lowland forests

Zone (latitudes)	Total area (million km^2^)	Current mean GPP (kg m^−2^ year^−1^)	Future mean GPP (kg m^−2^ year^−1^)	Current mean MCB (year^−1^)	Future mean MCB (year^−1^)	Current mean AGB (kg m^−2^)	Future mean AGB (kg m^−2^)	Mean AGB change (kg m^−2^)	Total C change (Pg)
Boreal (55° N–90° N)	11.0	0.70	0.92	0.11	0.14	10.06	11.59	1.52	8
North temperate (23.5° N–55° N)	15.9	1.58	1.73	0.18	0.22	22.36	18.33	− 4.03	− 30
Tropics (23.5° S–23.5° N)	21.7	3.21	2.95	0.31	0.35	37.67	22.18	− 15.50	− 160
South (90° S–23.5° S)	3.2	2.33	2.42	0.21	0.24	41.66	35.15	− 6.51	− 10
World	51.7	2.13	2.11	0.22	0.26	27.37	19.55	− 7.82	− 193

Current values were estimated based on the climate of 1970–2000 and future values based on a projected climate for 2081–2100

MCB (maintenance cost per unit biomass) had a similar global pattern as GPP based on the current climate (Fig. 1c). However, increasing temperatures increased MCB in all regions, and all MCB classes moved towards the poles (Fig. 1d). As with GPP, the ratio of MCB in the tropics to MCB in the boreal dropped, but only slightly, i.e. from 2.8 to 2.5.

As AGB was modelled based on the ratio of GPP to MCB (Eq. 1), and both increase towards the equator, we expected a more complex global pattern. Tropical, subtropical and temperate forests were estimated to have the highest AGBs, with a clear decreasing trend towards the north but also towards the central and eastern parts of the continents both in Eurasia and North America, which have more continental climates, i.e. more seasonal temperature variations (Fig. 1e). The highest AGBs were located in regions elevated to at least a few hundred metres above sea level in the tropics and subtropics, such as central Borneo but also in maritime mid-latitudes such as North island of New Zealand. Interestingly, as northern temperate humid lowland forests had a more continental climate than forests in the zone south, located at a similar distance from the equator, the mean AGB for the tropics was significantly higher than for northern temperate regions but somewhat lower than for the south (Table 1).

The increasing temperatures led to a dramatic drop in the AGB of the tropics but to an increase in the boreal (Fig. 1g). The resulting AGB pattern was more even than that based on the current climate (Fig. 1f). However, as the mean drop of 41% in AGB in the tropics is so significant relative to the modest increase of 15% in the boreal, the overall global change is strongly negative (Table 1).

The estimated decrease in humid lowland forest C from the current to the future was 193 Pg of C (Table 1). The drop in tropical AGB caused 83% of this global decrease. The period during which this estimated change would happen is uncertain, but assuming a century of change, the annual emission rate of 1.93 Pg year^−1^ can be compared e.g. to an increase of 4.9 Pg year^−1^ in atmospheric C in recent years [47].

Our results were surprisingly robust to changes in the parameters, parameter bounds or data selection criteria in the earlier study [24] that we tested (Additional file 1). However, considerable variation still occurred. For example, the global AGB decrease estimated at 7.82 kg m^−2^ actually ranged from 5.93 to 10.08 kg m^−2^ in the sensitivity analysis. As expected, much of this variation was caused by uncertainty in the tropics, large areas of which will have future climates not currently found on Earth.

## Discussion

We applied a simple physiological model that previously successfully explained current global AGB variation based on monthly temperatures [24, 25], to estimate the impacts of rising temperatures on the AGB of humid lowland forests. Our approach was based on focusing on GPP and ecosystem respiration, which are equal by definition in a steady-state old-growth forest. Duffy et al. [48] similarly focused on GPP and ecosystem respiration to study forest C budgets, but their focus on a 30-day moving window fails to acknowledge that the timing within the annual cycle and temperature dependence of heterotrophic respiration do not influence how much biomass living trees can maintain. Instead, we divided ecosystem respiration by a power of AGB, called this MCB, and assumed it to depend on temperature.

We predict a dramatic drop in global AGB. Perhaps surprisingly, our results cannot be compared directly to any given published study. Many articles report future biomass changes due to changes in regimes of natural [49] or anthropogenic [50] disturbances, but we focused on forests that are not disturbed. The Earth system models report changes from all global change drivers instead of focusing just on temperature [51], as we did.

Our aim was not to predict the impact of rising temperatures on forests to be used directly in global C balance predictions but instead to present the results of our approach as basis for further discussions. Therefore, the simplicity of our model should be viewed as an advantage, as its pros and cons are easier to comprehend than those of more complex methods. Based on our approach, AGB decreases if the ratio of GPP to MCB decreases (Eq. 2), and MCB can be viewed as the maintenance cost of a unit mass of a given biomass type per unit of time. As both GPP and MCB are dependent on temperature, their ratio obtains its maximum at a given temperature. This temperature is 16.4 °C assuming no diurnal temperature variation and a given sun path [24, 25]. Their ratio decreases in warmer temperatures, as MCB increases faster than GPP, while GPP decreases faster than MCB in colder temperatures. Seasonal temperature variation decreases AGB when mean annual temperature is close to this optimum, as this variation inherently shifts temperatures away from the optimum. In addition, fitting of these physiological models included parameters that cut GPP and increase MCB proportionally to the temperature difference to the previous month due to imperfect acclimation [24]. The importance of seasonal temperature variation in our approach explains why many statistical studies based on mean annual temperature fail to explain much of the global AGB variation, and why southern temperature forests that are more maritime have higher AGB than northern [21].

The simple modelling approach that we used successfully predicted global AGB patterns [24, 25] compared to other modelling approaches, but failed to explain certain more local findings. Based on our energetic principles, when nights only are considered, colder temperatures should allow more rapid growth and greater maximal size, as GPP is zero when light is not available because MCB decreases with decreasing temperature. In contrast to this, small trees have grown faster during warmer nights in an outdoor experimental setup with tropical pioneer seedlings [52]. However, this should perhaps be regarded as an exception because the proportion of biomass and growth of small pioneers in full light is very small, and indeed the growth-boosting effect of warmer nights disappeared with larger trees [12]. It seems likely that warming nights enhance the growth of small seedlings if their growth is not limited by available energy but by the speed of new tissue synthesis that increases with increasing temperature, or perhaps the large diurnal temperature variations are challenging for several biochemical processes. A second set of findings that is difficult to explain with our energetic approach comes from elevational gradients. Studies on elevational gradients starting from the lowlands in the tropics have revealed both decreasing and increasing biomasses towards higher elevations [17], even though when focusing only on temperatures and energetics, biomasses should peak in mean annual temperatures of approximately 10–15 °C, depending on the diurnal temperature variation. This corresponds to an elevation of approximately 2 km close to the equator [53]. Globally, the tallest trees have been shown to grow in this thermal climate in maritime regions in the mid-latitudes. Reasons for this potentially include the lack of huge trees in most tropical mountain ranges, but these reasons remain partially unclear [54]. A third shortcoming in the energetic approach that we applied relates to its fundamental assumptions. It is not difficult to see how e.g. a patch of evergreen species has both a smaller GPP and MCB than a neighbouring patch of deciduous species with the same AGB. However, based on the relative tight fit of the GPP and MCB models [24, 25], this does not seem to cause much variation and is even less likely to cause significant bias.

Despite its limitations, our approach based on GPP and MCB is overall likely to be the optimal methodology if this level of simplicity and physiology is pursued. However, even in a hypothetical situation in which only temperatures were to change, our predictions should not be taken as estimates of AGB in 2081–2100. Most of the original field measurements according to which the physiological models were parameterised were conducted a couple decades later [55–57] than the climate data we used [58]. Based on this, our current AGB is from the turn and first decades of the twenty-first century, and our future AGB would be relevant at the beginning of the twenty-second century. However, this would give an overly exact image of our predictions, as complex time lags may blur the real picture. These lags may drastically depend on whether the future dominant tree species are already dominant in the forest, are present but rare, or need to migrate to the location. When species-poor communities face dramatic climatic changes, significant migration may be needed and AGB may be significantly depressed after the early mortality of the original tree population. Such processes may be the cause of the current increased mortality [13] if sustaining the earlier AGB is not possibly energetically.

Exact AGB predictions become even more challenging when other global change factors, such as the fertilising effect of increasing atmospheric CO_2_ and N deposition, are also considered. It could be that the negative temperature impact and positive fertilisation impact roughly compensate each other in tropical lowland rainforests [59]. However, proportionally stronger impacts should not be assumed to still balance out, as a positive impact far above the evolutionary history of the tree may not be physiologically significantly more beneficial than a smaller impact, while a negative impact below the range in evolutionary history could be disastrous. Further complications include changes in precipitation and transpiration that are challenging to forecast, and trees may be able to transpire less thanks to increasing atmospheric CO_2_ [60] but may even transpire more to cool their leaves down in certain conditions [61]. Water balance may also influence biomasses via disturbances, especially fires, which may override other mechanisms in the boreal [62].

We estimated emissions of nearly 200 Pg of C due to the warming temperatures. However, this estimation was assuming that all humid lowland area was old-growth forest, which is far from the reality. Natural disturbances, such as fires, have influenced a large proportion of area especially in the boreal, and much of the area unsuitable for forest growth is covered by waterbodies or waterlogged soils, or is disturbed by humans through logging or more severely through agriculture or settlements.

Numerous avenues exist for future studies following these same principles (Eq. 2). Complexity could be increased by taking actual land uses and their changes into account and comparing them with observed patterns [63], by including arid and high areas or by incorporating other mechanisms such as the CO_2_ fertilisation effect. Alternatively, this same energetic approach (Eq. 2) can be used to understand succession unlike the more traditional way to understand AGB (Eq. 2). Temperature-based understanding of succession would open new possibilities for e.g. optimising wood production and storing C globally in both the current and future climates.

## Conclusions

Based on our approach, we predict a severe drop in the AGB of tropical forests and some decrease in the AGB of temperate forests. In contrary, we expect an increase in the AGB of boreal forests thanks to a longer and warmer growing season. However, this increase is minuscule per unit area and especially globally when multiplied by its area, when compared to decreases in the warmer regions. Scientists have had overly optimistic views concerning the impact of rising temperatures on AGB, as they have focused too much on small plants that are easier to monitor and on productivity despite its variation not explaining current global AGB variation [64].

## Supplementary Information

**Additional file 1.** Sensitivity of results relative parameters, parameter bounds and data selection criteria.

## Data Availability

The study is based on data sets on GPP and AGB published with several articles and available for easy reference as appendix of article [24]. The climate data that we used is available in www.worldclim.org/data/worldclim21.html. Future climate was based on the Coupled Model Intercomparison Project Phase 6 (CMIP6), providing information for Intergovernmental Panel on Climate Change (IPCC)’s 6th Assessment Report (AR6), processed for eight global climate models: BCC-CSM2-MR, CNRM-CM6-1, CNRM-ESM2-1, CanESM5, IPSL-CM6A-LR, MIROC-ES2L, MIROC6, and MRI-ESM2-0. Future scenario was SSP245, the update of RCP4.5 based on SSP2, representing the medium part of the range of plausible future pathways. We filtered the lowlands by topography of limit to elevations below 1 km by Global Multi-resolution Terrain Elevation Data (www.usgs.gov/centers/eros/science/usgs-eros-archive-digital-elevation-global-multi-resolution-terrain-elevation). This was based on the mean elevation in the whole grid, resampled from 30 s to 10 min.
